# Functionalized Surface Coatings for Rigid Contact Lenses

**DOI:** 10.3390/jfb15060154

**Published:** 2024-06-05

**Authors:** Roeya Refaei, Kyueui Lee, Goun Amy Lee, Paul Demian, Fouad El Mansouri, Phillip B. Messersmith, Mouad Lamrani, Mohamed Khaddor, Nabil Allali

**Affiliations:** 1 Laboratory of LAMSE, Faculty of Sciences and Techniques of Tangier, Abdelmalek Essaâdi University, B.P. 416, Tangier 90000, Morocco; roeya.refaei@etu.uae.ac.ma (R.R.); mkhaddor@uae.ac.ma (M.K.); 2 Department of Chemistry and Green-Nano Materials Research Center, Kyungpook National University, Daegu 41566, Republic of Korea; kyueui@knu.ac.kr; 3 Bioengineering and Materials Science and Engineering Departments, University of California, Berkeley, CA 94720, USA; up202009635@up.pt (G.A.L.); philm@berkeley.edu (P.B.M.); 4 Menicon R&D Innovation Centre, Menicon Co., Ltd., Nagoya (Japan), Geneva Branch, 1205 Geneva, Switzerland; p.demian@meniconrd.com (P.D.); m.lamrani@menicon.com (M.L.); 5 Research Team: Materials, Environment and Sustainable Development (MEDD), Faculty of Sciences and Techniques of Tangier, Abdelmalek Essaâdi University, B.P. 416, Tangier 90000, Morocco; 6 Menicon Co., Ltd., 21-19, Aoi 3, Naka-ku, Nagoya 460-0006, Japan

**Keywords:** polyphenols, peptoid, coating, antifouling

## Abstract

This research evolves into a comparative study of three different phenolic composites as coatings for rigid contact lenses, with a particular emphasis on enhancing their antifouling properties and hydrophobicity. The primary layer, comprised of diverse phenolic compounds, serves as a sturdy foundation. An exclusive secondary layer, featuring synthetic peptoids, is introduced to further minimize biofouling. Validated through X-ray photoelectron spectroscopy, the surface analysis confirms the successful integration of the polyphenolic layers and the subsequent grafting of peptoids onto the lens surface. The efficacy of the proposed coatings is substantiated through protein adsorption tests, providing definitive evidence of their antifouling capabilities. This research employs a nuanced assessment of coating performance, utilizing the quantification of fluorescence intensity to gauge effectiveness. Additionally, contact angle measurements offer insights into wettability and surface characteristics, contributing to a comprehensive understanding of the coating’s practicality.

## 1. Introduction

Over the past few decades, contact lenses (CLs) have demonstrably supplanted traditional spectacles as the preferred method of vision correction, primarily due to their enhanced comfort and unobtrusive nature [1]. However, widespread adoption has been encumbered by several key challenges, including ocular discomfort [2], protein deposition on the lens surface [3], and suboptimal wettability [4]. These factors can adversely impact both lens performance and visual acuity. In response, the emergence of surface modification techniques has marked a transformative juncture in the field of CL technology, fundamentally reshaping the CL experience and offering promising avenues for improved ocular health and visual outcomes [5].

Contact lenses fall into two main categories based on their material: hard lenses (including rigid gas permeable, or RGP) and soft lenses (SCLs) [6]. Though RGP lenses require an initial adjustment period due to their interaction with the eye, as Ichijima and Cavanagh point out [7], their significantly higher oxygen permeability and tear exchange make them a compelling choice. For both soft and RGP lenses, the quality of the lens is determined by several factors, including permeability [8], wettability [9], and protein adsorption [10].

Building upon recent advancement, F. Behboodi-Sadabad and H. Zhang [11] explored the possibility of harnessing the power of naturally occurring phenol molecules [12]. Phenols, when exposed to the skin or eyes, are highly irritating for next-generation contact lens coatings, opening doors to novel functionalities and enhanced wearer comfort. These phenols exhibit promising properties for surface modification due to their inherent adhesiveness [13], combined with their antioxidant [14], anti-inflammatory [15], and antibacterial capabilities [16]. As a monomer, phenols have the potential for negative effects, which may cause a range of adverse health effects [17], but as a polyphenol, show a promising biocompatibility [18].

**Figure 1:**
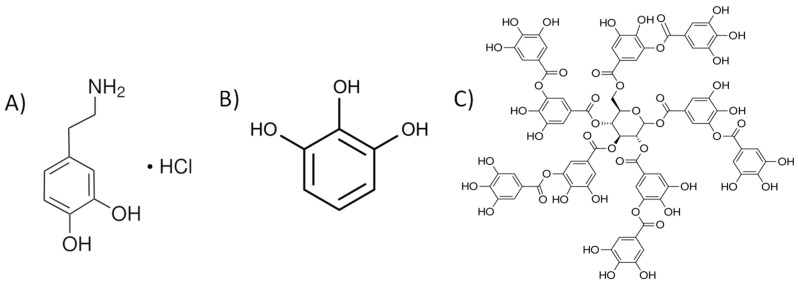
Structures of phenolic compounds used in this research: (**A**) dopamine hydrochloride, (**B**) pyrogallol, and (**C**) tannic acid.

Drawing inspiration from mussel adhesive chemistry, Haeshin et al. [19] showcased the power of polydopamine illustrated in Figure 1A (PDA) [20], opening doors to a wide range of potential applications across various fields. Notably, polydopamine coatings can be readily formed on diverse surfaces via a simple dip-coating procedure [21].

Furthermore, the adhesive properties of certain plant-derived phenols [22] present additional avenues for surface modification. Polypyrogallol (PPG) as shown in Figure 1B [23] and polytannic acid illustrated in Figure 1C (PTA) [24] have shown promise as coating precursors, as their adhesiveness is enhanced through oxygen exposure in mildly alkaline environments. This enables the formation of robust coatings on diverse substrates [25].

**Figure 2:**
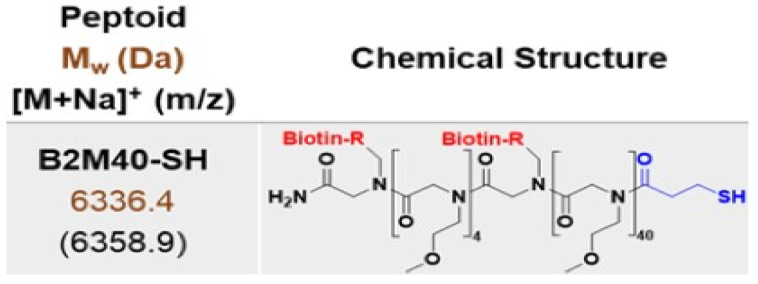
Description of the peptoid that is used for grafting on phenolic coating.

The potential of these coatings can be further amplified. Concepcion et al. [26] propose amplifying their potential by adding a secondary layer of peptoids (Figure 2) [27], paving the way for tailored functionalities and optimized performance.

Resembling peptides [28], featuring a synthetic nitrogen backbone and diverse side chains, peptoids offer exceptional adaptability and stability in biological settings [29], often surpassing their natural counterparts [30].

This versatility, coupled with the inherent adhesiveness of the underlying PDA, PPG, and PTA layer [31], paves the way for the creation of highly multifunctional surfaces. This makes peptoids ideal candidates for a wide range of applications, extending beyond contact lens technology.

This study aimed to achieve three key objectives: improve antifouling properties, enhance wettability, and demonstrate the viability of a functional ophthalmic lens. Our approach involved a two-step surface functionalization technique on RGP contact lenses in an alkaline solution, overcoming the inherent difficulty of chemically modifying fluorine-containing hydrophobic lenses. A phenolic coating was first applied as a primer, followed by the grafting of peptoids onto the surface. This approach was chosen to leverage the combined advantages of each material: the adhesive properties of the phenolic coating, the antifouling, and the protein-repelling capabilities of peptoids.

To provide a comprehensive understanding of the modified lens surfaces, we employed a multifaceted analytical approach. X-ray photoelectron spectroscopy (XPS) enabled detailed characterization of the phenolic and peptoid layers, elucidating their structure, integration, and potential synergistic interactions. Additionally, FITC-conjugated albumin assays provided insights into protein adsorption dynamics, while water angle measurements quantified the enhanced hydrophilicity of the modified surfaces.

## 2. Materials and Methods

### 2.1. Chemicals and Materials

All chemicals used in our protocol were obtained from Sigma Aldrich (Milwaukee, WI, USA). The peptoid was synthesized in the Berkeley laboratory using established protocols [27,32]. X-ray photoelectron spectroscopy (XPS) measurements were performed at the Berkeley University facility on an Omicron ESCALAB system (Omicron, Taunusstein, Germany). Green fluorescence from FITC-conjugated albumin was measured using a Synergy H1 instrument (Agilent Technologies, Inc., Winooski, VT, USA) with a 96-well plate. Contact angle measurements were conducted using a Drop Shape Analyzer DSA-25.

### 2.2. Phenolic Coating

The methods employed in this study were adapted from previous works issued by the group of Prof. P. B. Messersmith for dopamine [19], pyrogallol, and tannic acid [33]; these established protocols ensured reliable experimental procedures.

Our experiment started with cutting an RGP into four pieces, which were then cleaned five consecutive times with deionized water to remove any possible contamination. After cleaning, the pieces were dried with N_2_ gas.

Each lens piece underwent a dip-coating process for 3.5 h, applying distinct coating conditions. (1) A 1 mg/mL quantity of dopamine hydrochloride was dissolved in 0.1 M bis-tris at a pH 8.5. (2) For pyrogallol, 1 mg/mL was dissolved in a solution of 0.1 M bis-tris, containing 0.1 M MgCl_2_, at a pH of 7.0. (3) Tannic acid (1 mg/mL) was dissolved in 0.1 M bis-tris and 0.6 M NaCl, at a pH of 7.8.

- The pH adjustments were made using NaOH.

### 2.3. Peptoid Grafting

Polyphenol-coated contact lenses, prepared as described earlier, acted as the substrate for subsequent peptoid grafting. The lenses were submerged in a solution containing 0.5 mg/mL of peptoid dissolved in a 10 mM tris buffer at pH 8.5, maintained at a temperature of 25 °C. The immersion process continued overnight with continuous shaking at 50 rpm.

Post-grafting, the lenses were subjected to a series of deionized water washes (5 times) to remove unbound peptoid molecules. Finally, the lenses were dried thoroughly using a stream of N_2_ gas.

### 2.4. Verifying the Coating with XPS

Survey and high-resolution XPS spectra were collected on an Omicron ESCALAB (Omicron, Taunusstein, Germany) configured with a monochromated Al Kα (1486.8 eV) 300-W X-ray source, 1.5 mm circular spot size, a flood gun to counter charging effects, and an ultrahigh vacuum (<10^−8^ Torr).

### 2.5. Protein Adsorption Tests on Polyphenol-Coated Contact Lenses

FITC-conjugated albumin was dissolved in HEPES buffer (10 mM) containing 150 mM NaCl, reaching a concentration of 3 mg/mL.

The samples underwent an incubation process at 37 °C for 18 h with continuous shaking at 50 rpm. Following the incubation period, the lenses were washed extensively with deionized water (5 times). The lenses were then carefully dried using a stream of N_2_ gas.

### 2.6. Quantification of the Protein Adsorption Using Microplate Reader

To quantify the fluorescence intensities of adsorbed proteins on the substrates, we used a microplate reader. Each lens was put into a separate well of a 96-well-plate.

The wavelength of 490 nm (theoretical excitation wavelength of FITC) was applied to excite the FITC-conjugated albumin. Since the area of the contact lens was varied; the intensity of the measured fluorescence was divided by the calculated area for a precise comparison. The area was calculated by the software program version 1.54i of ‘ImageJ’.

### 2.7. Measuring the Wettability of the Surface by Static Contact Angle

Surface water was removed from the sample with KIMTECH wipe paper. The sample was put on a sample holder and a drop of DI water (2 μL) put on the top of the lens. The calculation of the contact angle was performed using ADVANCED software (Kruss, Berlin, Germany) taking into account the curvature of the sample.

## 3. Results

### 3.1. XPS Examination of Surface Functionalization

**Figure 3:**
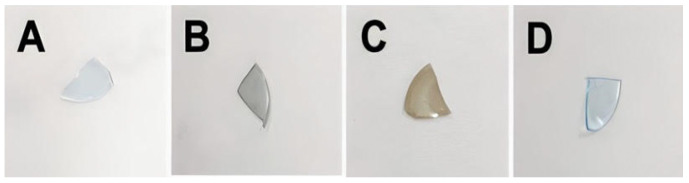
Optical Images before and after the phenolic coatings: (**A**) Bare, (**B**) PDA, (**C**) PPG, (**D**) PTA.

Using a cutter blade, the contact lens was divided into four pieces, and each resulting piece underwent dip-coating in a phenolic solution (Figure 3). Subsequently, distinct changes in coloration were observed in the segments coated with PDA and PPG. Interestingly, the transparency of the contact lens coated with PTA was comparable to the bare lens.

**Figure 4:**
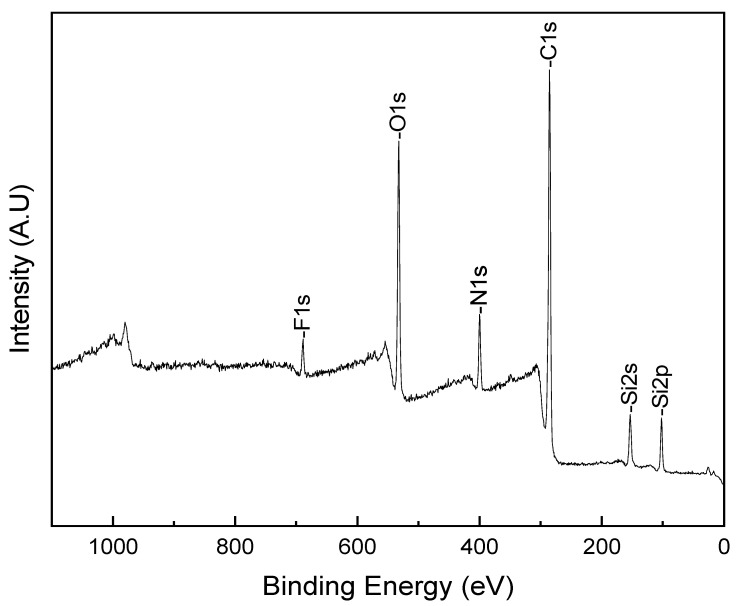
Survey peaks of a bare contact lens.

The XPS spectrum (Figure 4) demonstrates the chemical elements present in our bare substrate: C1s (carbon), N1s (nitrogen), O1s (oxygen), Si2s, Si2p (silicon), and F1s (fluorine). The intensity of the peaks informs us of quantitative information and the appearance of the elements present in the substrate. As fluorine is more exposed to the surface compared to silicone and nitrogen, this is the reason why the F peak is our reference for the coating.

We observed that the dominant element is the C1s, which makes our bare substrate primarily composed of carbon, followed by oxygen, silicon, nitrogen, and lastly, fluorine, as shown in Table 1, by atomic percentage (At%).

#### 3.1.1. Analyzing the Surface after PDA Coating and Peptoid Grafting

**Figure 5:**
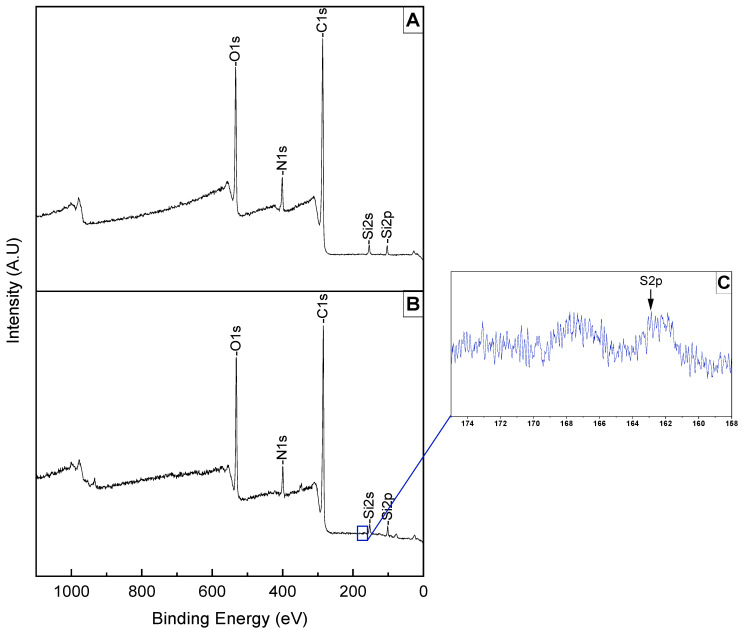
Survey peaks of (**A**) PDA coating on an RPG lens, (**B**) peptoid grafting, and (**C**) high-resolution S2p peak.

To confirm the PDA coating and the peptoid grafting on the surface of the RGP, we applied the XPS surface analysis, and our survey peaks showed a difference in the intensity of the peaks. The first observation, after the PDA coating, was the disappearance of the ‘fluorine peak’ and the decrease of the ‘silicon peaks’; these changes were realized by the change in the thickness of the RGP, reflecting the sequential presence of these elements within the contact lens, which implies that the surface was successfully coated (Figure 5A).

We also noted a change in the intensity of peaks in the spectrum, which is confirmed via the At% (Table 2). An increase in the intensity of C1s and O1s is due to the PDA structure which is rich in carbon and oxygen; the decreased intensity of N1s is a result of the coating process, indicating that despite the presence of nitrogen in the PDA structure, the nitrogen originally present in the contact lens was effectively covered by the PDA coating.

To verify the grafting of peptoids into the surface of the contact lens, we analyzed high-resolution sulfur peaks from the sample (Figure 5C), since the peptoid can be distinguished from the phenolic compounds by the presence of sulfur in the molecular structure (Figure 2—one from the thiol group, the others from biotins). In the XPS spectrum (Figure 5B) we noticed a minor trace of sulfur between 158–174 eV.

#### 3.1.2. Analyzing the Surface after PPG Coating and Peptoid Grafting

For the PPG coating, the first thing we noticed was the disappearance of the N1s peak; this is due to the thickness of the coating covering it. The decrease in the Si peaks was also the result of the coating.

**Figure 6:**
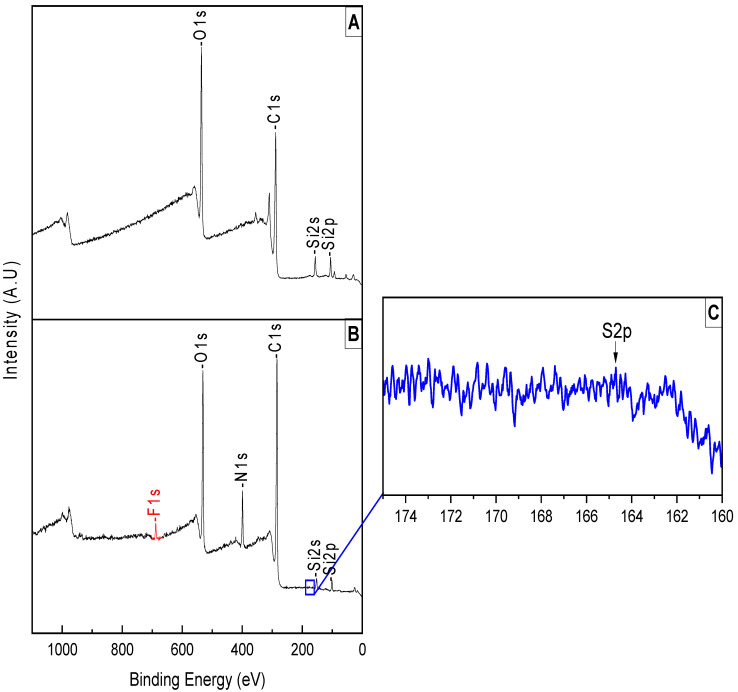
Survey peaks of (**A**) PPG coating on an RGP lens, (**B**) peptoid grafting, and (**C**) high-resolution S2p peak.

We observe in Figure 6A a decrease in C1s and O1 peak intensity, resulting from the coating of PPG.

However, after the grafting of the peptoid, we observed that the F1s peak was exposed, indicating that the PPG coating was ripped off in a relatively basic (pH 8.5) condition. This also explains the reappearance of the N1s peak; we have a small increase in the peak compared to the bare contact lens (Table 1), due to the nitrogen in the peptoid structure. Furthermore, we see an increase in the Si peak; this is due to the peptoid grafting into the surface. The sulfur was detected at a marginally detectable concentration (Table 3).

#### 3.1.3. Analyzing the Surface after PTA Coating and Peptoid Grafting

**Figure 7:**
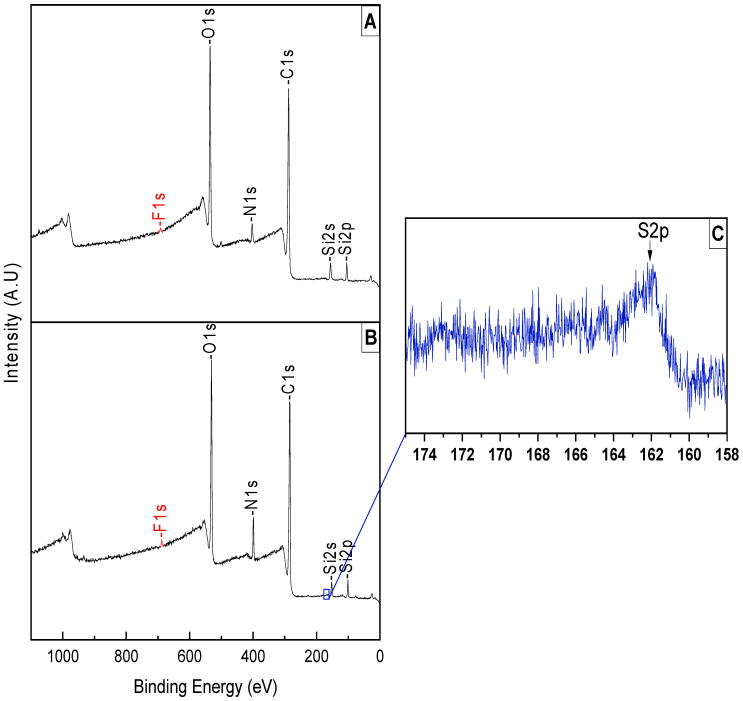
Survey peaks of (**A**) PTA coating on RGP lens, (**B**) peptoid grafting, and (**C**) high-resolution S2p peak.

The PTA coating was confirmed via XPS surface analysis as shown in Figure 7A. The analysis indicated an unnoticeable decrease in a C1s peak, but a noticeable increase in O1s; this is due to the structure of PTA being full of oxygen. N1s, Si2p, Si2s, and F1s peaks showed a decrease in their peaks, indicating that the PTA did not fully coat the surface, since we can observe the trace of these peaks.

Following the grafting of the peptoid (Figure 7B), we notice an increase in the N1s peak, as well as the C1s peak, because of the peptoid structure. We also notice the decrease of the O1s, Si2p, and Si2s peaks as shown in Table 4, which means the peptoid covered those elements in the contact lens.

There is a small increase in the F1s peak.

### 3.2. Protein Adsorption Tests on Polyphenol-Coated Contact Lenses

#### 3.2.1. Green Fluorescence from FITC-Conjugated Albumin

**Figure 8:**
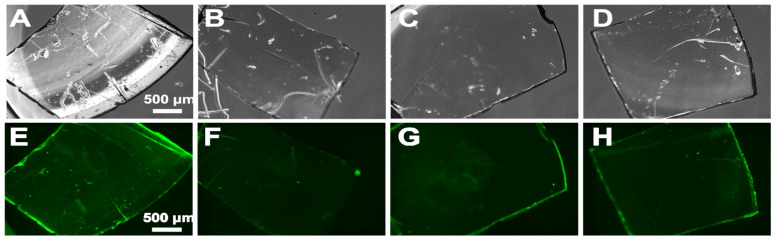
Optical (**A**–**D**) and fluorescence (**E**–**H**) images of each lens after FITC-albumin adsorption on (**A**,**E**) Bare substrate; (**B**,**F**) PDA/peptoid-coated substrate; (**C**,**G**) PPG/peptoid-coated substrate; (**D**,**H**) PTA/peptoid-coated substrate.

Green fluorescence from FTIC-conjugated albumin was visualized by a confocal microscope (Figure 8), with an exposure time of 1s; green fluorescence was observed from the bare substrate (Figure 8A,E). Otherwise, the contact lens with the peptoids on the polydopamine surface rarely showed green fluorescence (Figure 8B,F). This demonstrates the antifouling effect on the peptoid-functionalized PDA surface. Even though we do not know if the peptoids were conjugated, we also tested the PPG-coated sample (Figure 8C,G). Fluorescence was observed, but it was much dimmed compared to the bare substrate, which might be due to the remaining peptoids on the surface. The sample with peptoids on PTA also exhibited an antifouling effect on proteins, but the effect was seemingly comparable to the PPG-coated sample (Figure 8D,H).

#### 3.2.2. Quantification of Protein Adsorption Using a Microplate Reader

Since the area of the contact lens was varied (Table 5), the intensity of as measured fluorescence was divided by the calculated area for the precise comparison. The area was calculated by the software program ‘ImageJ’.

**Figure 9:**
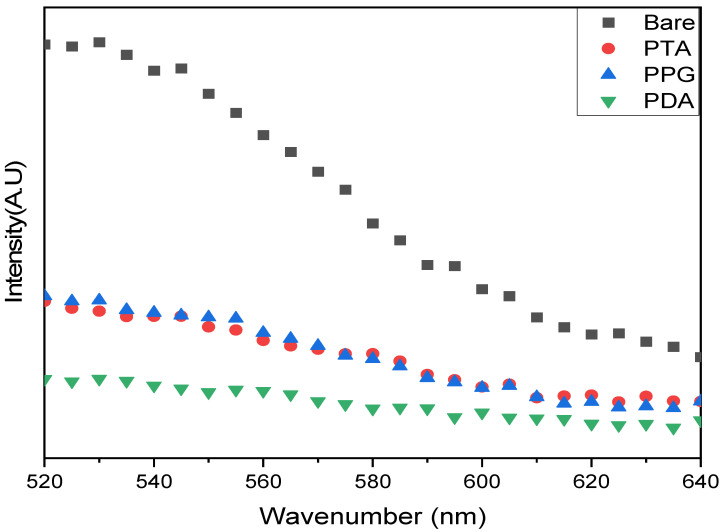
Quantified fluorescent intensities from each peptoid-modified polyphenol-coated substrate.

In the subsequent quantification, fluorescent intensities from each coated and grafted substrate were systematically measured (Figure 9). The relative intensity of fluorescence measured from peptoid-modified polyphenols is greatly reduced compared with the substrate (bare contact lens only). According to the data, the peptoid grafted on the polydopamine showed the strongest antifouling effect.

### 3.3. Water Contact Angle Measurements

#### 3.3.1. ‘Bare’ vs. ‘PDA’ vs. ‘PDA w/Peptoid’ (*n* = 5)

**Figure 10:**
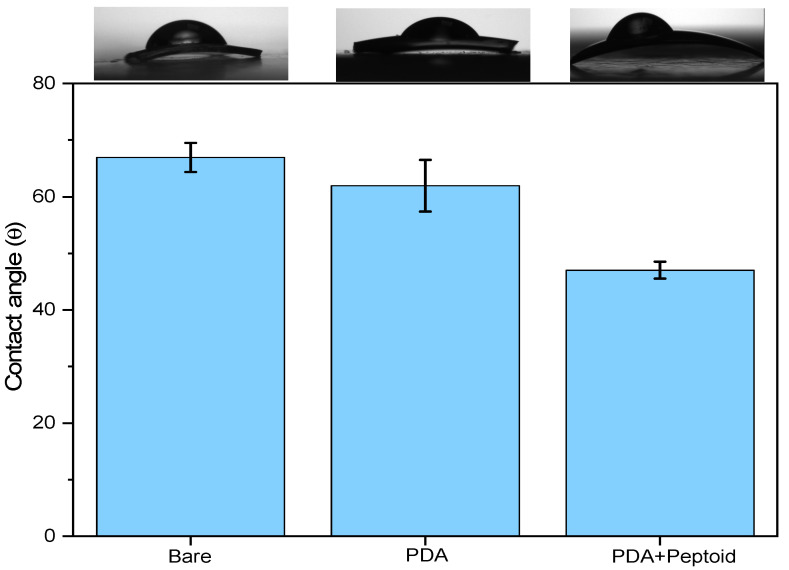
Variation of water contact angles: on the bare contact lens, PDA coating on the bare contact lens, and peptoid grafting on the PDA.

As we previously investigated by XPS (Figure 5A), PDA can be successfully deposited on the contact lens surface, and the result is double-checked by confirming the increased hydrophilicity of the contact lens after the coating process (Figure 10). The bare contact lens was at 67 ± 2.5°, and after the coating, to 62 ± 4.5°. The hydrophilicity of the substrate was further increased after the peptoid grafting at 47 ± 1.5°, indicating that the surface modification using peptoid was successful.

#### 3.3.2. ‘Bare’ vs. ‘PPG’ vs. ‘PPG w/Peptoid’ (*n* = 5)

**Figure 11:**
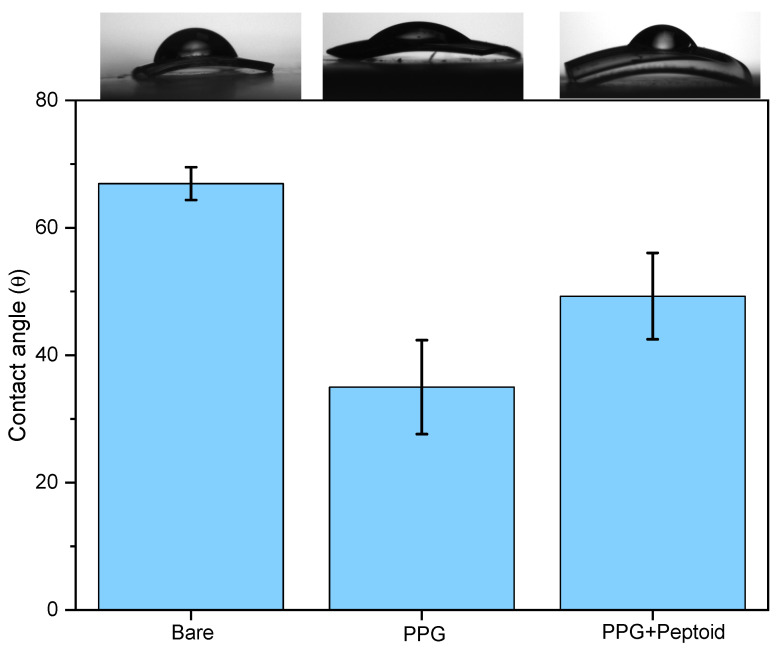
Variation of water contact angles: on the bare contact lens, PPG coating on the bare contact lens, and peptoid grafting on the PPG.

Hydrophilicity was increased after the PPG coating at 35 ± 7.4° (Figure 11), compared to the PDA coating. This is due to the relatively high portion of oxygenated groups inside the molecular structure of pyrogallol (Figure 1C). After the peptoid grafting, the contact angle was increased to 49. 3 ± 6.8°, which is comparable to the value of the peptoid-grafted PDA surface. This indicates that the peptoid grafting was also successful on the PPG coating, even though we could not observe a strong peptoid signal (S2p) from the XPS results (Figure 6C).

#### 3.3.3. ‘Bare’ vs. ‘PTA’ vs. ‘PTA w/Peptoid’ (*n* = 5)

**Figure 12:**
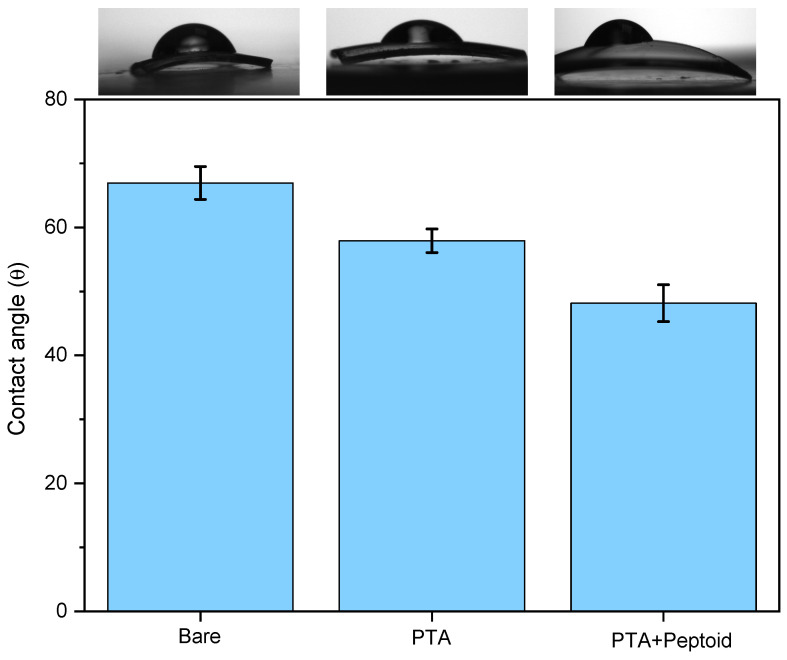
Variation of water contact angles: on the bare contact lens, PTA coating on the bare contact lens, and peptoid grafting on the PTA.

The hydrophilicity of the contact lens sequentially increased after two surface modification steps, PTA coating followed by peptoid-grafting as confirmed in Figure 12, which once again demonstrates that both surface modifications were successful (also previously confirmed by the XPS-high resolution S2p, Figure 7C). After the PTA coating at 58 ± 1.8° and the peptoid grafting, the contact angle was decreased to 48.2 ± 2.9°.

## 4. Discussion

Herein, we demonstrate that three different phenolic compounds can be successfully applied to a hydrophobic contact lens. Considering that the color of the lens should be minimized after coating, in our experiment, PTA was the most suitable technique for surface functionalization, as it did not result in any color change. The peptoid was also successfully grafted onto PDA and PTA surfaces, and for PPG, we need to adjust the pH level.

The unexpected reduction in fluorescence intensity on polyphenol-coated substrates highlights the need for diverse protein adsorption assessment methods.

After the peptoid grafting on each polyphenolic surface, all the contact angles were decreased from the original contact angle value of 67 ± 2.6° (bare), and the values are almost comparable: the values ranged from 47° to 49°. From the results, we can notice two things: surface modification by peptoid grafting was successfully done on all three different phenolic coatings (PDA, PPG, PTA). Due to the intrinsic property of the relatively hydrophilic peptoid, the surface becomes more hydrophilic.

Future endeavors should prioritize optimizing peptoid grafting for increased density, improved wetting, and decreased protein adsorption, offering promising advancements in contact lens surface modification techniques.

## 5. Conclusions

In conclusion, our study successfully grafted peptoids onto contact lenses using phenolic coatings, achieving an effective antifouling effect. PTA emerged as a promising option, demonstrating minimal color change and reasonable antifouling. While PDA-coated lenses exhibited minimal antifouling, the color changes were noticeable. PPG, despite color and stability challenges, shows potential with optimization. Further refinement of coating conditions, thickness, and peptoid composition may enhance overall performance. Post-peptoid grafting, contact angles uniformly decreased, indicating improved hydrophilicity for all three coatings.

## Figures and Tables

**Table 1:** The atomic percentage of the elements on the bare substrate.

Element	C1s	O1s	Si2p	Si2s	N1s	F1s
At%	64.8	18.1	8.4	7.6	7.2	1.4

**Table 2:** Atomic percentage after the 1st coating of PDA and grafting of peptoid as 2nd coat.

Element	C1s	O1s	Si2p	Si2s	N1s	F1s	S2p
PDA_At%_	70.8	20.7	2.6	1.5	6.5	0	0
PDA-Peptoid_At%_	70.2	21.7	2.6	1.5	5.6	0	<0.1

**Table 3:** Atomic percentage after the PPG coating and the peptoid grafting.

Element	C1s	O1s	Si2p	Si2s	N1s	F1s	S2p
PPG_At%_	60.9	30.06	7.8	6.4	0	0	0
PPG-Peptoid_At%_	67.9	21.9	2.3	1.2	6.8	1	<0.1

**Table 4:** Atomic percentage after the PTA coating and the peptoid grafting.

Element	C1s	O1s	Si2p	Si2s	N1s	F1s	S2p
PTA_At%_	63.4	27.9	5.1	3.8	3.3	0.3	0
PTA-Peptoid_At%_	64.9	25.9	3.3	2.6	6.2	0.5	<0.1

**Table 5:** Area of each contact lens.

	BARE	PDA	PPG	PTA
Area of Lens	6.8 mm^2^	7.2 mm^2^	6.9 mm^2^	6.3 mm^2^

## Data Availability

The original contributions presented in the study are included in the article, further inquiries can be directed to the corresponding author.
